# Anti-GBM disease after nephrectomy for xanthogranulomatous pyelonephritis in a patient expressing HLA DR15 major histocompatibility antigens: a case report 

**DOI:** 10.5414/CNCS108594

**Published:** 2015-11-02

**Authors:** Emma O’Hagan, Tamara Mallett, Mairead Convery, Karl McKeever

**Affiliations:** 1. Royal Belfast Hospital for Sick Children, Pediatric Renal Unit, Belfast, Northern Ireland

**Keywords:** anti-GBM, antiglomerular, HLA DRB15, nephrectomy, pediatric

## Abstract

Antiglomerular basement membrane (anti-GBM) antibody disease is uncommon in the pediatric population. There are no cases in the literature describing the development of anti-GBM disease following XGP or nephrectomy. We report the case of a 7-year-old boy with no past history of urological illness, treated with antimicrobials and nephrectomy for diffuse, unilateral xanthogranulomatous pyelonephritis (XGP). Renal function and ultrasound scan of the contralateral kidney postoperatively were normal. Three months later, the child represented in acute renal failure with rapidly progressive glomerulonephritis requiring hemodialysis. Renal biopsy showed severe crescentic glomerulonephritis with 95% of glomeruli demonstrating circumferential cellular crescents. Strong linear IgG staining of the glomerular basement membranes was present, in keeping with anti-GBM disease. Circulating anti-GBM antibodies were positive. Treatment with plasma exchange, methylprednisolone, and cyclophosphamide led to normalization of anti-GBM antibody titers. Frequency of hemodialysis was reduced as renal function improved, and he is currently independent of dialysis with estimated glomerular filtration rate 20.7 mls/min/1.73 m^2^. Case studies in the adult literature have reported the development of a rapidly progressive anti-GBM antibody-induced glomerulonephritis following renal surgery where patients expressed HLA DR2/HLA DR15 major histocompatibility (MHC) antigens. Of note, our patient also expresses the HLA DR15 MHC antigen.

## Introduction 

Antiglomerular basement membrane (Anti-GBM) disease has an estimated incidence of ~ 0.5 – 1.0 per million per year in the adult population and is less common in children [1]. It is characterized by rapidly progressive crescentic glomerulonephritis, which is historically associated with a poor renal outcome and end-stage renal disease [2, 3, 4]. The outcome in adults has improved following the introduction of plasmapheresis together with immunosuppressive drugs [3, 5]. Recent pediatric cases have also shown good clinical recovery following early aggressive immunosuppressive treatment including plasmapheresis [6, 7]. 

There are many theories as to the etiology of anti-GBM disease. Recent case studies in the adult literature have reported the development of a rapidly progressive anti-GBM antibody induced glomerulonephritis 3 – 12 months after lithotripsy where patients expressed HLA DR2/HLA DR15 major histocompatibility (MHC) antigens [8, 9]. The HLA DR15 antigen is strongly associated with anti-GBM disease in Caucasian adults [10]. We can find no cases in the pediatric literature describing anti-GBM disease following renal surgery, specifically nephrectomy. We present the case of a 7-year-old boy who developed anti-GBM disease following nephrectomy for xanthogranulomatous pyelonephritis (XGP). 

## Case report 

**Figure 1.**
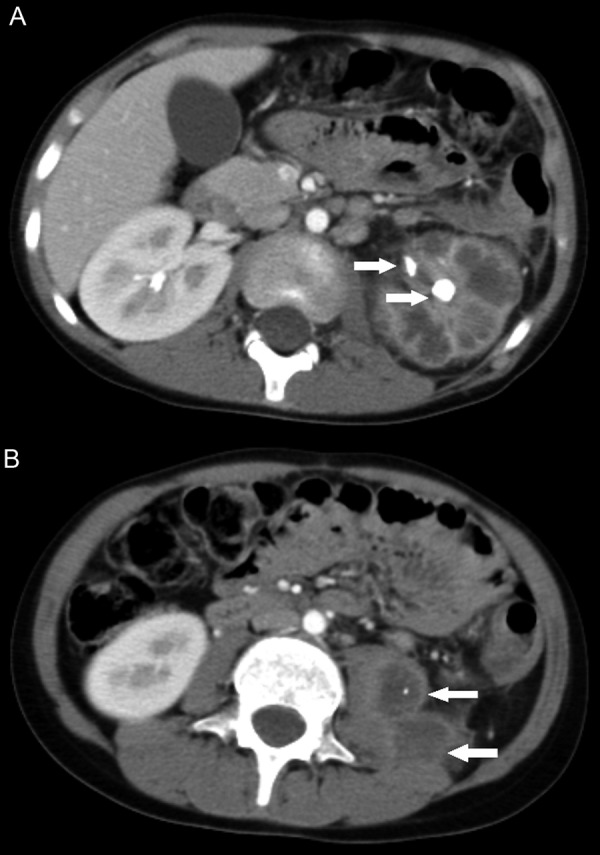
Axial CT source images. A: At the level of the renal hila. The swollen left kidney enhances poorly following intravenous contrast administration. There is calyceal distension and overlying cortical thinning. High attenuation calculus material is seen in the renal pelvis (white arrows). There is inflammatory stranding in the pararenal fat. B: Just inferior to the left kidney. Well-defined, low attenuation lesions are seen in both the enlarged left psoas and iliacus muscles (white arrows); the former contains a fleck of calcification. The rim of both lesions enhances with the administered intravenous contrast. Appearances are consistent with left-sided xanthogranulomatous pyelonephritis and secondary abscess formation. The psoas abscess contains an extruded calculus.

**Figure 2.**
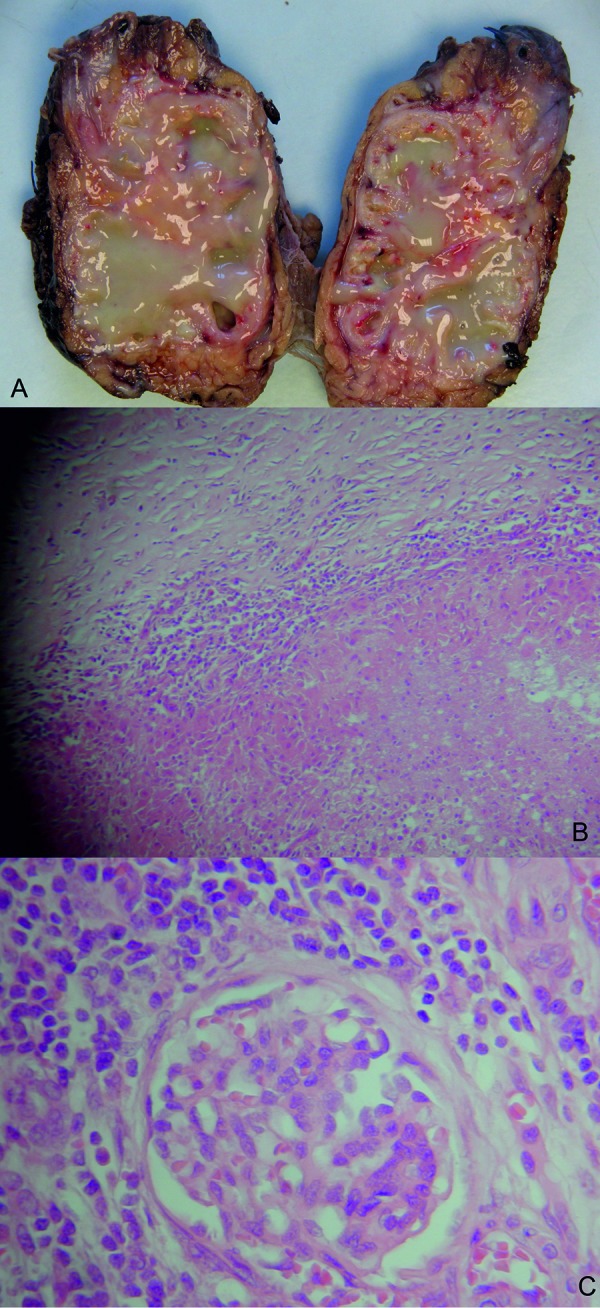
Gross nephrectomy specimen. A: Gross nephrectomy specimen – dilated renal pelvis filled with a large of amount of yellow pus-like material displaying regional areas of hemorrhage. B: Light microscopy of renal parenchyma with Hematoxylin and Eosin stain. Inflammatory infiltrate with foamy histiocytes and neutrophil clusters. Ziehl-Neelsen and auramine staining were negative for tuberculosis. C: Light microscopy of normal area of tissue from nephrectomy specimen showing no evidence of anti-GBM disease.

A 7-year-old Caucasian male presented to urology services with symptoms of left flank pain and pyrexia, and a 6-month history of intermittent back pain. On examination, he was normotensive with left flank tenderness, and urinalysis was positive with microscopic hematuria, proteinuria, and leucocytosis. Inflammatory markers were elevated with C-reactive protein (CRP) 156 mg/L and hemoglobin (Hb) low at 10 g/dL. A CT abdomen was in keeping with unilateral XGP (Figure 1). Following 14 days of antimicrobial therapy, he underwent a left open nephrectomy with good clinical response. Histopathology confirmed XGP, with Proteus mirabilis isolated on microbiological culture (Figure 2). Postoperative contralateral renal ultrasound was normal and renal function preserved, creatinine pre-operatively was 0.57 mg/dL, with creatinine 0.53 mg/dL, and estimated glomerular filtration rate (eGFR), calculated by the modified Schwartz method, of 102 mls/min/1.73 m^2^ on discharge. 

Three months later, the child represented with a 24-hour history of cola-colored urine and reduced urine output. On examination, he was pyrexial and clinically dehydrated. He was normotensive with no abdominal tenderness or edema. Urinalysis was positive for microscopic hematuria and proteinuria with sterile culture. Renal function was impaired with creatinine 1.7 mg/dL and eGFR 32 mL/min/1.73 m^2^. Hb was 11.5 g/dL, white cell count 1.8 × 10^9^/L with neutropenia of 0.55 × 10^9^/L. C-reactive protein was raised at 150 mg. C3 was mildly raised, but C4 was normal. Cytoplasmic antineutrophil cytoplasmic antibodies (C-ANCA), perinuclear anti-neutrophil cytoplasmic antibodies (P-ANCA). and antinuclear antibodies were negative, and antistreptolysin O titre (ASOT) was positive at 800 units/L. Immunoglobulins were normal, and viral and microbial screening was negative. Renal ultrasound scan was normal, excluding XGP in the contralateral kidney. 

A working diagnosis of poststreptococcal glomerulonephritis was made, and intravenous antimicrobials (co-amoxiclav) commenced with careful fluid management. The patient’s clinical condition continued to deteriorate with persistent pyrexia, oliguria, and declining renal function. By day 3 of admission, eGFR had fallen to 21.9 mL/min/1.73 m^2^. Renal biopsy was deferred at this stage due to the associated risks of a single kidney. A day-7 repeat ultrasound showed a single enlarged hyper-echogenic right kidney consistent with acute glomerulonephritis (GN) or pyelonephritis. IV antimicrobial treatment was broadened with ciprofloxacin and meropenem to cover for organisms associated with XGP; however, repeat blood and urine cultures were sterile. CT of the abdomen excluded recurrent XGP. Inflammatory markers remained elevated with a continual decline in eGFR to 11.1 mL/min/1.73m^2^ on day 12 of admission. The patient developed clinical manifestations of acute renal failure with hypertension, pulmonary edema, and fluid overload necessitating hemodialysis. 

**Figure 3.**
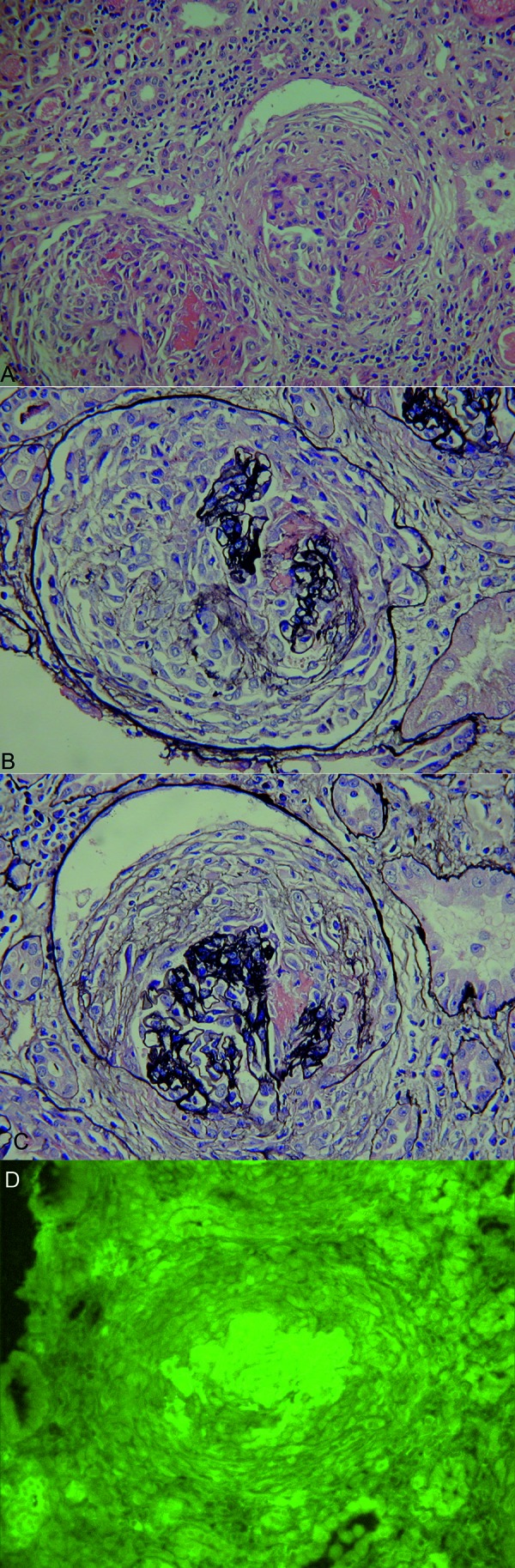
Histopathological images. A: Hematoxylin and Eosin stain showing diffuse circumferential crescentic and necrotizing injury of the glomerulus, characteristic of anti-GBM disease. B and C: Methenamine silver stain shows remnants of the glomerular basement membrane (GBM) surrounded by a cellular crescent occupying bowman’s space and focal fibrinoid necrosis. D: Immunofluorescence showing strong linear IgG of the GBM characteristic of anti-GBM disease. The glomerulus is compressed by a cellular crescent (not visible on immunofluorescence).

No antihypertensive medication was required. A trial of high-dose methylprednisolone was commenced at 500-mg IV daily for 3 days followed by 60 mg/m^2^ daily oral prednisolone for presumed rapidly progressive glomerulonephritis. Despite this, creatinine continued to climb to a peak of 6.5 mg/dL (eGFR 8.5 mL/min/1.73 m^2^) and the decision was made for renal biopsy. Histology revealed strong linear IgG deposition along the GBM with greater than 95% of glomeruli demonstrating crescents and evidence of acute tubular necrosis (Figure 3). Anti-GBM antibodies confirmed the diagnosis of anti-GBM disease with titers of > 8 AI (normal range 0 – 0.9 AI). The patient’s tissue typing confirmed the presence of the HLA DR15 antigen. 

Treatment was commenced with plasmapheresis and oral cyclophosphamide at 3 mg/kg/day for 8 weeks. High-dose steroid therapy and thrice weekly hemodialysis was continued. Nineteen plasma exchanges were performed over 28 days with normalization of anti-GBM antibodies to 0.4 AI. Following cessation of plasma exchange, 4 further hemodialysis sessions were required for fluid and blood pressure control. Renal function gradually improved, with serum creatinine at time of last dialysis (day 78 following admission) 2.76 mg/dL and eGFR 20 mL/min/1.73 m^2^. Cyclophosphamide was temporarily discontinued due to neutropenia and subsequently reintroduced at alternate-day intervals, with no adverse effect, to a cumulative dose of 168 mg/kg. 

The patient is now 12 months post completion of plasma exchange for anti-GBM disease and remains dialysis free. He continues on a weaning dose of alternate day prednisolone. Anti-GBM titers remain normal at 0.2 AI/mL. Renal function is stable with serum creatinine of 2.1 mg/dL, eGFR 26 mL/min/1.73 m^2^. 

## Discussion 

Anti-GBM disease has an estimated incidence of 0.5 – 1.0 per million per year in the adult population and is much rarer in children [1]. It is characterized by the formation of autoantibodies against the noncollagenous domain of the α3 chain of type IV collagen of the GBM [6, 11]. Pathologically, crescentic changes typically affect most glomeruli [3]. The clinical presentation in adult patients is a rapidly progressive glomerulonephritis, often accompanied by pulmonary hemorrhage. The lung complications however are rarely observed in children, perhaps because of their association with smoking. 

The underlying mechanisms leading to the production of anti-GBM antibodies is unknown. Speculative etiological factors include exposure to viral and bacterial infections, toxins, such as hydrocarbons and smoking, penicillamine treatment, ANCA-associated GN, and membranous nephropathy. There is also a strong HLA association, with over 80% of individuals carrying HLA DR15 or DR4 alleles [12]. A number of case reports have described the development of anti-GBM disease in adult patients following lithotripsy, suggesting that antigen release or exposure from damage to the kidney may initiate anti-GBM disease in susceptible individuals [8, 9, 13, 14, 15, 16, 17, 18, 19]. In these cases, the affected individuals presented between 3 and 12 months after lithotripsy. Significantly, our patient also expresses the HLA DR15 antigen and presented with rapidly progressive anti-GBM disease 3-months post nephrectomy. We hypothesize that in this case, XGP caused disruption of the GBM in a pro-inflammatory environment, leading to the development of autoimmunity to the Goodpasture antigen. This mechanism would be consistent with that proposed after lithotripsy or following ANCA-associated GN or membranous nephropathy. 

Diagnosis of anti-GBM disease is confirmed on renal biopsy by IgG deposition along the basement membrane. In most cases, circulating anti-GBM antibodies are also positive. Anti-GBM antibody testing should be considered in all cases of rapidly progressive GN irrespective of patient age or renal history, and, in retrospect, should have been performed earlier in our case. Diagnosis in this case was obscured by previous presentation with XGP and a positive ASOT. The presence of a solitary kidney with associated risks of renal biopsy further compounded the diagnostic difficulties. 

Most cases of anti-GBM disease in pediatric patients have acute presentations with diffuse glomerular disease, including severe fibrinoid necrosis along with cellular crescent formation, with subsequent glomerular scarring leading to end-stage renal disease [6, 11, 20, 21, 22, 23, 24, 25]. More recent reports, where diagnosis and commencement of plasmapheresis has been prompt, have led to better outcomes [7, 26, 27, 28]. In our case, plasmapheresis, together with immunosuppressive drugs, has led to a marked improvement in renal function. Eleven months post presentation, the patient remains dialysis independent with a stable GFR of 26 mL/min/1.73 m^2^. 

We believe this to be the first reported case of a child with anti-GBM antibody disease following XGP and nephrectomy. This case adds strength to the hypothesis that GBM disruption in the presence of the HLA DR4 or 15 antigen may trigger the development of anti-GBM disease. It adds to the limited number of published cases of children with anti GBM disease and the growing body of evidence suggesting a much improved renal outcome with plasmapheresis in combination with immunosuppression. 

Anti-GBM disease should be considered in the differential diagnosis of rapidly progressive glomerulonephritis in all patients, irrespective of age, especially if presentation follows recent renal injury or surgery. Early diagnosis of anti-GBM antibody disease and treatment with plasmapheresis and immunosuppression can lead to a favorable renal outcome. 

## Acknowledgments 

The authors would like to thank the following for their contribution to this case: Dr. Mary O’Connor, Consultant Nephrologist Royal Belfast Hospital for Sick Children, Dr. Declan O’Rourke, Consultant Histopathologist, Royal Group of Hospitals, Belfast, and Dr. Annie Paterson, Consultant Pediatric Radiologist, Royal Belfast Hospital for Sick Children, Professor Charles Pusey, Consultant Nephrologist, Imperial College London. 

## Conflicts of interest 

There are no conflicts of interest to declare.
